# Fermentation characteristics, chemical composition and microbial community of tropical forage silage under different temperatures

**DOI:** 10.5713/ajas.18.0085

**Published:** 2018-07-26

**Authors:** Dongxia Li, Kuikui Ni, Yingchao Zhang, Yanli Lin, Fuyu Yang

**Affiliations:** 1 College of Animal Science and Technology, China Agricultural University, Beijing 100193, China; 2 Beijing Sure Academy of Biosciences, Beijing 100085, China

**Keywords:** Bacterial Diversity, Fermentation, Silage, Tropical Forage

## Abstract

**Objective:**

In tropical regions, as in temperate regions where seasonality of forage production occurs, well-preserved forage is necessary for animal production during periods of forage shortage. However, the unique climate conditions (hot and humid) and forage characteristics (high moisture content and low soluble carbohydrate) in the tropics make forage preservation more difficult. The current study used natural ensiling of tropical forage as a model to evaluate silage characteristics under different temperatures (28°C and 40°C).

**Methods:**

Four tropical forages (king grass, paspalum, white popinac, and stylo) were ensiled under different temperatures (28°C and 40°C). After ensiling for 30 and 60 days, samples were collected to examine the fermentation quality, chemical composition and microbial community.

**Results:**

High concentrations of acetic acid (ranging from 7.8 to 38.5 g/kg dry matter [DM]) were detected in silages of king grass, paspalum and stylo with relatively low DM (ranging from 23.9% to 30.8% fresh material [FM]) content, acetic acid production was promoted with increased temperature and prolonged ensiling. Small concentrations of organic acid (ranging from 0.3 to 3.1 g/kg DM) were detected in silage of white popinac with high DM content (50.8% FM). The microbial diversity analysis indicated that *Cyanobacteria* originally dominated the bacterial community for these four tropical forages and was replaced by *Lactobacillus* and *Enterobacter* after ensiling.

**Conclusion:**

The results suggested that forage silages under tropical climate conditions showed enhanced acetate fermentation, while high DM materials showed limited fermentation. *Lactobacillus* and *Enterobacter* were the most probable genera responsible for tropical silage fermentation.

## INTRODUCTION

With the rapid economic development in tropical areas, the demand for animal products is increasing greatly. It is well known that tropical forage is sufficient or even available in excess for animal feed during the fast growing season, whereas there can be limited supply in winter when the forage grows slowly. This has become the main factor limiting the improvement of animal product quantity and quality in these regions. Therefore, a suitable storage technique for green forage is necessary to cover these shortages in the tropics [1].

Ensiling has been regarded as a common way for preserving green forage in temperate areas, because of its long shelf life, good palatability and high nutrition [2]. However, the application of ensiling techniques in tropical areas has been limited when compared with that in temperate regions. Previous studies reported that the fermentation pattern of tropical forage silages might differ from that of temperate silages [3]. The main difference is that lactic acid is the major fermentation product for temperate silages, whereas acetic acid is often regarded as the primary fermentation product in tropical silages [4]. However, little information is available on the microbial community associated with the promotion of acetic acid fermentation. Characterizing the microbial population and activity might help to understand the unique fermentation conditions and improve the silage quality in the tropics.

Temperature is one of the crucial factors impacting silage quality. Different temperature conditions can affect the activity of microorganisms in silages, which can also influence the fermentation process [5]. In general, the ensiling temperature is around 28°C in temperate areas, whereas the temperature can reach up to around 40°C in tropical regions. The impacts of high temperature (>37°C) have been studied on various temperate forage silages such as alfalfa, corn and other crops. Some studies have found that hot conditions (around 40°C) lowered lactic acid production, whereas acetic acid production was enhanced [4,6,7]. However, tropical silage fermentation under high temperature has rarely been studied, despite the fact that high temperature might be an important environmental factor in the tropics.

Hainan is the southernmost province of China with the typical tropical climate of warm to hot and moist throughout the year. King grass (*Pennisetum purpureum*×*P. americana*), paspalum (*Paspalum plicatulum*), white popinac (*Leucaena leucocephala*), and stylo (*Stylosanthes guianensis*) are widely cultivated forage species with high yield and good palatability for livestock in this region. Increasing efforts are devoted to improving their preservation for animal production maintenance. This study was designed to evaluate the natural fermentation characteristics of these tropical forage silages under different temperatures (28°C and 40°C).

## MATERIALS AND METHODS

### Silage preparation

The experiment was conducted at the experimental base of the Tropical Genetic Resource Institute of the Chinese Academy of Tropical Agriculture Science, Dan Zhou, Hainan, China (109°30′E, 19°30′N). Four forages (king grass [*Pennisetum purpureum*×*P. americana* cv. Reyan No.4], paspalum [*Paspalum plicatulum* cv. Reyan No.11], white popinac [*Leucaena leucocephala* cv. Reyan No.1] and stylo [*Stylosanthes guianensis* cv. Reyan No.2]) were harvested at the vegetative stage in July, 2016.

The harvested materials were wilted overnight and chopped by a forage cutter set to about 2 cm in length. Silages were prepared using a small-scale system: 200 g of each type of forage material were packed into plastic bags (N-9, Asahi Kasei Co., Ltd., Tokyo, Japan), and the air was removed using a vacuum sealer. The silos were stored at low temperature (28°C in incubator) and high temperature (40°C in incubator) for 30 and 60 days. Each treatment of the four forages was conducted in triplicate.

### Microbial enumeration, organic acid and chemical composition analysis

Silage samples (10 g) were blended with 90 mL of sterilized water and serially diluted from 10^−1^ to 10^−5^ in sterilized water. Bacterial counts were estimated for lactic acid bacteria (LAB) using de Man, Rogosa and Sharpe agar (incubated anaerobically). Yeasts and molds were enumerated on spread plates of potato dextrose agar. The plate cultures were incubated at 30°C for 48 h (media were obtained from Beijing Aoboxing Bio-tech Co., Ltd., Beijing, China). Microbial counts were converted to log_10_ and presented on a fresh material (FM) basis.

Silage pH, ammonia nitrogen (NH_3_-N) and organic acid contents were determined from water extracts. The pH was measured using a glass electrode pH meter (PHS-3C, INESA Scientific Instrument, Shanghai, China). The NH_3_-N content was analyzed using the method described by Broderick and Kang [8]. Lactic acid, acetic acid, propionic acid and butyric acid were analyzed using high-performance liquid chromatography (LC20A, Shimadzu, Ltd., Tokyo, Japan).

Dry matter (DM) content was determined by drying the material in an oven at 65°C for 48 h. Crude protein (CP) was analyzed according to the guidelines provided by Latimer [9]. Neutral detergent fiber (NDF) and acid detergent fiber (ADF) were determined using the methods described by Van Soest et al [10]. The water-soluble carbohydrates (WSC) content was determined using the anthrone method [11].

### Analysis of bacterial diversity through high-throughput sequencing of metagenomic DNA

The four pre-ensiled forages and silage samples were added to a 20×volume of sterilized phosphate-buffered saline (pH 7.4), and DNA extraction was performed as described by Ni et al [12]. The V3–V4 region of the bacterial 16S ribosomal RNA gene was amplified by polymerase chain reaction (PCR) (95°C for 2 min followed by 25 cycles of 95°C for 30 s, 55°C for 30 s, 72°C for 30 s and a final extension at 72°C for 5 min) using the primers 338F (ACTCCTACGGGAGGCAGCAG) and 806R (GGACTACH VGGGTWTCTAAT). To minimize PCR bias, triplicate PCR reactions were conducted for each sample, and mixtures of the three PCR products were used for measuring the DNA concentration and sequencing [12,13]. The DNA samples were paired-end sequenced (2×250) on an Illumina MiSeq platform at Majorbio Bio-Pharm Technology Co., Ltd. (Shanghai, China). For quality-control purposes, any sequences that contained mismatches and ambiguous reads in the primers were removed.

Operational taxonomic units (OTUs) were clustered with a similarity cutoff of 97% using UPARSE (version 7.1, http://drive5.com/uparse/), and chimeric sequences were identified and removed using UCHIME. The taxonomy of each 16S rRNA gene sequence was analyzed using the ribosomal database project (RDP) Classifier (http://rdp.cme.msu.edu/) against the SILVA (SSU115) 16S rRNA database with a confidence threshold of 70%. The alpha-diversities of the samples, mainly the Shannon index, Chao richness estimator, and Good’s coverage, were calculated using Mothur (version 1.30.1, http://www.mothur.org/wiki/Classify.seqs). Taxonomic classification at the genus level was performed using the RDP algorithm to classify the representative sequences of each OTU [14].

### Statistical analysis

Data on fermentation characteristics, chemical composition and microbial community composition after ensiling were analyzed with a completely randomized design with a 2×2 (ensiling days [D]×storage temperature [T]) factorial treatment structure. The means were then compared for significance using the Duncan’s multiple range method. All statistical analysis was performed using the general linear model procedure in SAS 9.0 (SAS Institute, Cary, NC, USA, 2002). Significance was declared at p<0.05 unless otherwise noted.

## RESULTS

### Characteristics of four tropical forages before ensiling

The chemical composition and microbial community composition of the four tropical forages prior to ensiling are shown in Table 1. White popinac had the highest DM content (50.8% FM), followed by stylo (30.8% FM), paspalum (25.9% FM) and king grass (23.9% FM). The CP content ranged from 66.8 to 264 g/kg DM; the highest and lowest values were found in white popinac and paspalum, respectively. The NDF and ADF contents ranged from 456 to 716 and from 255 to 455 g/kg DM, respectively. The WSC contents were not >30.0 g/kg DM. Overall, the LAB population were around 5.0 log colony forming units (cfu)/g FM and the molds and yeasts were around 3.0 log cfu/g FM.

### Fermentation characteristics of four tropical forage silages

Fermentation characteristics of the four tropical silages are shown in Table 2. Ensiling days and storage temperature showed significant effects on pH and lactic acid content for king grass and stylo silages (p<0.001). The pH decreased gradually with increased temperature and prolonged ensiling for these two forages, while the lactic acid content showed the opposite trend. Similar results were found for paspalum and white popinac, although the effects were not all significant. When storage was prolonged up to 60 days, the pH values of the four tropical silages at 40°C, were the lowest, and the lactic acid content was the highest for each forage species. Acetic acid content increased gradually over the ensilage for all four tropical silages (p<0.001); the highest acetic acid content was obtained for king grass silage (38.5 g/kg DM) after 60 days ensiling at 40°C. The highest content of propionic acid (16.5 g/kg DM) and butyric acid (4.7 g/kg DM) was observed after 60 days at 40°C in paspalum and king grass silage, respectively. For white popinac silage, lactic acid content ranged from 1.6 to 3.1 g/kg DM, while the other three organic acid contents were all very low (<1.0 g/kg DM). NH_3_-N content increased with prolonged ensiling time (p<0.001) and ranged from 10.1 to 17.5 g/kg DM for king grass silages. There was no significant effect of storage temperature on NH_3_-N for these tropical silages. Similar to the results of organic acid contents, white popinac silage had the lowest NH_3_-N content (<1.0% total nitrogen).

### Chemical composition of four tropical forage silages

Chemical composition of the silages is shown in Table 3. DM content decreased with prolonged ensiling time for these four tropical silages (p<0.001), whereas there were no significant differences in NDF, ADF, and CP content during the ensiling. WSC content decreased after 30 days ensiling. The lowest WSC contents were found at 40°C after 60 days of ensiling for all silages.

### Microbial population of four tropical forage silages

The interaction of ensiling days and storage temperature had significant effects on the population of LAB by enumeration using culture-based approaches (p<0.001; Table 4). After 30 days ensiling, silages stored at 40°C had higher numbers of LAB than those at 28°C (p<0.05). However, the opposite phenomenon was observed after 60 days ensiling, the numbers of LAB of the four forage silages decreased at 40°C, whereas they generally increased at 28°C. A small number of molds (≤3.0 log cfu/g FM) were detected only in white popinac silage at low temperature after 30 days ensiling. Yeast was not detected in all silages.

### Bacterial community of four tropical forage silages examined by high-throughput sequencing of metagenomic DNA

High-throughput sequencing of 16S rRNA gene amplicons was conducted to comprehensively characterize bacterial communities in four tropical forage silages. A total of 766,028 bacteria sequences (approximately 38,000 sequences per sample) were obtained. After bioinformatics analysis, a total of 552,860 sequences were classified. As listed in Table 5, these sequences were clustered into a total of 5,502 OTUs at a 3% dissimilarity level. The OTUs ranged from 124 to 565 for each sample, except for fresh paspalum material with the maximum value of 1,055. The Chao index, which was utilized to estimate the number of OTUs, showed a similar trend to OTUs. The coverage values in all silages ranged from 0.978 to 0.999, suggesting that most of the bacterial communities were detected. The highest value of the Shannon index (the diversity index of the microbial communities) was obtained for white popinac (4.95) at high temperature after ensiling for 60 days.

**Figure 1:**
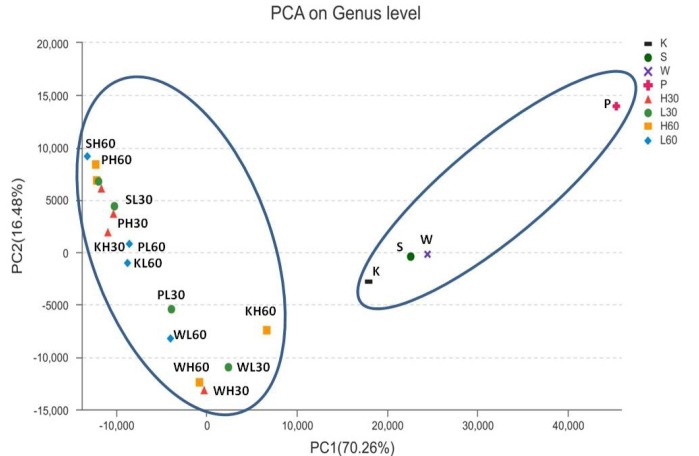
Principal component analysis of the four tropical forage samples on genus level. K, king grass; P, paspalum; W, white popinac; S, stylo; L, low temperature (28°C); H, high temperature (40°C); 30, 30 days; 60, 60 days.

The dynamic variance of bacterial community structure of four tropical forage silages was demonstrated by principal component analysis (PCA; Figure 1), where principal components 1 (PC1) and 2 (PC2) explained 70.26% and 16.48% of the total variance, respectively. The samples clustered into two groups: the four fresh tropical forages were in one group, while the silage samples were in the other.

**Figure 2:**
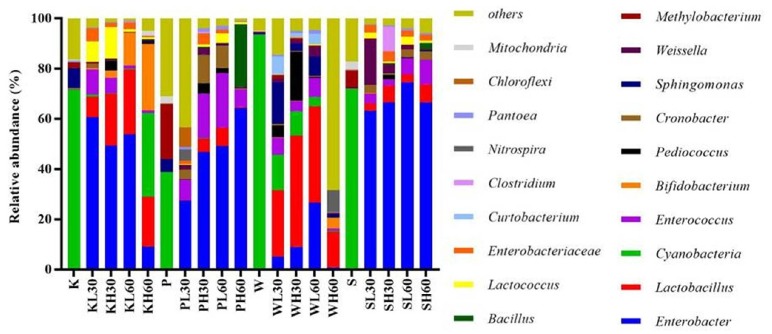
Genus-level microbiota analysis of the four tropical forages during ensiling. K, king grass; P, paspalum; W, white popinac; S, stylo; L, low temperature (28°C); H, high temperature (40°C); 30, 30 days; 60, 60 days.

Differences in bacterial community among the pre-ensiled forages and silages at the genus level with the threshold at >2.0% of the total, are shown in Figure 2. *Cyanobacteria* accounted for 71.7%, 38.9%, 93.2%, and 71.4% in pre-ensiled king grass, paspalum, white popinac and stylo, respectively; its proportion decreased to <1% after 30 days ensiling and increased to 33.4% after 60 days ensiling at 40°C in king grass silages. The opposite trend was found in white popinac, where the proportion of *Cyanobacteria* decreased gradually with prolonged ensiling and increased temperature; the proportion of *Cyanobacteria* was only 0.4% after 60 days ensiling at 40°C. For paspalum and stylo, *Cyanobacteria* decreased to marginal levels after ensiling. *Lactobacillus* and *Enterobacter* became the two predominant genera after ensiling, accounting for 11.6% and 33.7% of the total sequences, respectively. These two high abundance genera corresponded to the bacterial genus analysis in Table 5. The proportion of *Lactobacillus* ranged from 8.2% to 25.8% in king grass, 0.2% to 7.4% in paspalum, 14.2% to 44.4% in white popinac, and 2.9% to 7.2% in stylo; while the proportion of *Enterobacter* ranged from 9.2% to 60.7% in king grass, 27.5% to 64.4% in paspalum, 0.9% to 26.7% in white popinac and 63.3% to 74.6% in stylo. Many other LAB genera, i.e., *Enterococcus*, *Pediococcus*, *Weissella*, and *Lactococcus*, were also detected after ensiling (Figure 2). The proportions of *Enterococcus*, *Weissella*, and *Lactococcus* ranged from 1.0% to 21.7%, <0.1% to 18.3%, <0.1% to 12.6%, respectively, for these four silages. *Pediococcus* (1.8% to 19.6%) was mainly detected at 40°C after 30 days ensiling. *Bifidobacterium* was also detected in these silage samples, mainly in king grass silages, and it increased gradually with increased temperature and prolonged ensiling (0.4% to 26.5%).

## DISCUSSION

Although ensiling technique is quite mature and has been widely applied in temperate regions, it is not common in tropical areas, despite the high yield of tropical forages. Feeding silage is expected to improve the productivity of livestock in the tropics and increasing attempts are being carried out to develop a suitable ensilage process [15,16]. The differences in the internal factors and external environmental characteristics between tropical and temperate forages might result in distinct fermentation properties.

It is well known that pH and organic acid are important indicators reflecting microbial activity and silage fermentation. Previous studies reported that high temperatures (around 40°C) were unfavorable for LAB fermentation, leading to decreased organic acid concentration, increased pH and having a detrimental effect on the fermentation process for many temperate forage silages (i.e., alfalfa, wheat, corn) [5,6]. However, this was inconsistent with our finding on tropical silages. In the present study, pH was rapidly reduced for the silages at 40°C, especially for stylo, in which the pH decreased to 4.9 even with storage for only 30 days. When it was ensiled at 28°C, even after 60 days, the pH remained high at pH 5.2, which implied that fermentation for the tropical silage was more vigorous at 40°C than at 28°C.

It is well documented that lactate type fermentation is dominant in temperate forage silages. In the present study, acetic acid content was much higher than lactic acid in king grass, paspalum and stylo silages, and its content increased as the temperature increased and the ensiling was prolonged. Similar results were found in other studies that acetic acid was considered as the main acid responsible for preservation in tropical grass silages [1,3,4]. Nishino et al [17] reported that even if lactic acid production was observed when ensiling was initiated, prolonged ensiling was found to decrease the lactic acid level and increase the acetic acid level for tropical grass silages. This phenomenon suggested that different from the temperate forage silages, acetate type fermentation occurred in tropical silages and implied that the environmental conditions encountered in the tropics might affect the physiology and metabolism of the microorganisms involved in the silage fermentation.

The acetic acid production was higher at high storage temperature (40°C) when compared with that at low temperature (28°C); a similar trend was also demonstrated with guinea grass, maize and total mixed ration silages in temperate areas [7,18,19]. The efficient acetate fermentation at high temperature might be attributed to the thermodynamic implication, that high temperature facilitated the metabolism of acetic acid producing bacteria, like *Enterobacter* and hetero-fermentative LAB [7]. This might also account for the lower pH in silages of 40°C than in those of 28°C in the present study.

NH_3_-N was measured to follow potential protein breakdown that might have resulted from elevated temperature and prolonged ensiling time. Previous studies on temperate forages reported that high temperatures (around 40°C) usually led to detrimental effects on the fermentation process by facilitating the growth of proteolytic bacteria, and enhancing proteolysis, thereby exacerbating the NH_3_-N production. Our results were inconsistent with this trend, as high temperature exhibited no significant effects on NH_3_-N in the present study. A possible explanation for such differences might be that the high acetic acid content at high temperature inhibited some proteolytic microorganisms [20].

Microorganisms play an important role in silage fermentation; however, investigations on the microflora associated with tropical fermentation are rare. In this study, the microbial diversity of the four tropical forage silages was systematically analyzed by both culture-based and culture independent methods. The results indicated that numbers of yeasts and molds decreased during the ensiling process, especially for the silages stored at high temperature, where yeasts and molds were not detected after ensiling. The LAB counts were higher in silages stored at 40°C than at 28°C after 30 days ensiling, and this was consistent with the results of the high-throughput sequencing of metagenomic DNA, *Lactobacillus* was present at higher proportions when stored at 40°C than 28°C after 30 days ensiling in these four silages, which could account for the increases in the lactic acid content, and decreases in pH observed at high temperature. The dominance of *Lactobacillus* is desirable during silage fermentation as it is associated with increasing lactic acid concentration while reducing the pH [14,21,22]. However, it is hard to explain why the number of LAB decreased and the *Lactobacillus* proportion declined with increases in organic acid and the gradual decline in pH in silages at 40°C after 60 days ensiling. This might because the microflora evaluation was only performed at the beginning of the process and at 30 and 60 days of fermentation, whereas the fermentation products were accumulated throughout the fermentation process. Another possible explanation is that, in addition to *Lactobacillus*, some other bacterial genera could regulate the fermentation of tropical forages over long periods of ensiling.

Nishino et al [17] suggested an association between the appearance of *Enterobacter* and high acetic acid content in tropical guinea grass silages by denaturing gradient gel electrophoresis (DGGE) analysis, however, the abundance of this genus was unclear, as DGGE analysis is not quantitative. In the present study, the bacterial community analyzed by high-throughput sequencing confirmed this suggestion. *Enterobacter* was the predominant genus for the silages of king grass, paspalum and stylo with high acetic acid concentration. *Enterobacter* is a common genus found during silage fermentation and, various species of the *Enterobacter* genus were found in forage silages like corn, alfalfa, guinea grass and other forages in the tropics [15,23,24]. This genus can grow under anaerobic conditions and can protect themselves to overcome adverse conditions [25]. The main fermentation end-product of this genus is acetic acid, and the metabolic activity will be enhanced at high temperature [26,27]. Regarding the inhibitory activity of acetic acid [17], the relative abundance of *Enterobacter* might account for the rapid decrease in undesirable microorganisms during the ensiling process and might be responsible for the findings of Ostling and Lindgren [28] who reported that *Enterobacter* exhibited a positive effect on silages, with an unexpected increase in storage stability.

*Bifidobacterium* is well known as an important intestinal probiotic for humans and animals. This genus possesses a unique fructose-6-phosphate phosphoketolase pathway employed to ferment carbohydrates to acetic acid and lactic acid [29]. *Bifidobacterium* was identified in this study, mostly in king grass silages, and the abundance at high temperature was higher than that in low temperature silages; this might partly be related to the high contents of lactic acid (7.1 g/kg DM) and acetic acid (38.5 g/kg DM) that were found in king grass after 60 days ensiling at high temperature. Similar results were also reported for natural fermented alfalfa silage [30], that *Bifidobacterium* abundance increased with high acetic acid content throughout the ensiling period.

Some other LAB genera i.e., *Enterococcus*, *Weissella*, *Pediococcus*, and *Lactococcus* were found at considerable proportions in these silages; similar results were also found in other temperate forages, i.e., oat, soybean and grass silages [31–33]. Ni et al [33] suggested that cocci-shaped LAB were usually present in naturally fermented silages.

In this study, similar fermentation characteristics were observed for the tropical silages tested, except for white popinac, which produced few fermentation products. The chemical composition of white popinac showed that it had the highest DM content before ensiling with the highest residual WSC after ensiling, which might imply that the extent of fermentation was restricted. McDonald et al [2] reported that wilting is an important factor that directly influences the microflora. The wilting process selects for micro-organisms best able to survive under different moisture conditions. High DM reduces the activity of bacteria in silage and reduces the organic acid content for successful preservation. Muck et al [34] suggested that higher DM silage accumulated fermentation products slowly when compared with low DM samples, and that the rate and extent of fermentation during ensiling were limited by high DM content. Negative correlations between DM and acetic acid production was also demonstrated by Garcia et al [26] and Singh et al [35]. These previous results were consistent with our finding that white popinac silage had high DM and low concentrations of fermentation products, especially for acetic acid. However, high-throughput sequencing analysis found that white popinac silage contained a high proportion of genus *Lactobacillus*. The low content of organic acid and high pH of white popinac silage in this study might imply that the DM content affected the mechanism of the forage fermentation and that the quantity of LAB did not guarantee efficient tropical silage fermentation.

## CONCLUSION

The fermentation characteristics, chemical composition and microbial community of the four tropical forages (king grass, paspalum, white popinac, and stylo) under different temperatures (28°C and 40°C) of ensiling were studied. The results indicated that the four tropical forage silages are prone to enhanced acetic acid production with increased temperature and prolonged ensiling. The fermentation of white popinac with high DM was slow with low concentrations of fermentation products. *Cyanobacteria*, as the predominant microorganism before ensiling, was mainly replaced by *Lactobacillus* and *Enterobacter* after ensiling. These two genera were probably most responsible for the unique fermentation pattern in these four tropical forages.

## Figures and Tables

**Table 1:** Chemical and microbial composition of four tropical forages

Items	King grass	Paspalum	White popinac	Stylo
DM (% FM)	23.9	25.9	50.8	30.8
pH	6.7	6.2	6.1	6.0
CP (g/kg DM)	133	66.8	264	136
NDF (g/kg DM)	634	687	456	716
ADF (g/kg DM)	334	378	255	455
WSC (g/kg DM)	12.7	16.5	20.8	9.1
LAB (log cfu/g FM)	5.7	4.7	5.8	5.5
Molds (log cfu/g FM)	3.2	3.7	2.9	3.3
Yeasts (log cfu/g FM)	3.4	3.8	3.0	4.5

FM, fresh matter; DM, dry matter; CP, crude protein; NDF, neutral detergent fiber; ADF, acid detergent fiber; WSC, water-soluble carbohydrates; LAB, lactic acid bacteria; cfu, colony forming units.

**Table 2:** Fermentation characteristics of four tropical forage silages during ensiling

Items		30 days		60 days		SEM	p-value		
		
		28°C	40°C	28°C	40°C		D	T	D×T
King grass	pH	5.6^a^	5.2^b^	5.1^b^	4.8^c^	0.039	<0.001	<0.001	0.537
	LA	2.7^c^	5.4^b^	5.8^b^	7.1^a^	0.176	<0.001	<0.001	0.060
	AA	24.8^b^	25.6^b^	37.2^a^	38.5^a^	0.727	<0.001	0.508	0.866
	PA	1.0	1.3	1.3	1.1	0.096	0.779	0.685	0.192
	BA	2.2^b^	2.5^b^	2.9^b^	4.7^a^	0.128	<0.001	0.002	0.021
	NH_3_-N	10.1^b^	11.2^b^	15.5^a^	17.5^a^	0.391	<0.001	0.082	0.577
Paspalum	pH	5.2	5.2	5.2	5.1	0.034	0.145	0.755	0.361
	LA	1.1^b^	1.8^ab^	1.7^ab^	2.0^a^	0.103	0.059	0.039	0.438
	AA	13.5^b^	14.9^b^	21.6^a^	22.7^a^	0.739	<0.001	0.431	0.936
	PA	7.2^d^	9.2^c^	12.3^b^	16.5^a^	0.307	<0.001	0.001	0.113
	BA	1.4^b^	1.7^ab^	2.0^a^	2.0^a^	0.068	0.006	0.256	0.401
	NH_3_-N	3.9^b^	6.0^ab^	7.0^a^	7.0^a^	0.355	0.018	0.175	0.187
White popinac	pH	5.8^a^	5.6^b^	5.7^ab^	5.3^c^	0.027	0.003	<0.001	0.069
	LA	1.6^b^	2.3^ab^	1.9^b^	3.1^a^	0.142	0.107	0.009	0.418
	AA	0.3^c^	0.3^c^	0.9^a^	0.7^b^	0.016	<0.001	0.163	0.003
	PA	0.3	0.3	0.3	0.3	0.007	0.749	0.017	0.209
	BA	0.9	0.9	0.9	0.8	0.016	0.857	0.070	0.223
	NH_3_-N	0.9	0.8	0.8	0.9	0.054	0.881	0.777	0.444
Stylo	pH	5.4^a^	4.9^c^	5.2^b^	4.8^d^	0.019	<0.001	<0.001	0.709
	LA	1.9^c^	6.0^a^	4.0^b^	6.7^a^	0.121	<0.001	<0.001	0.028
	AA	7.8^b^	8.0^b^	10.5^a^	12.3^a^	0.279	<0.001	0.116	0.185
	PA	0.6^ab^	0.5^b^	0.7^a^	0.5^b^	0.015	0.314	0.008	0.788
	BA	1.6	1.4	1.5	1.6	0.029	0.401	0.398	0.061
	NH_3_-N	5.2	5.9	5.7	6.0	0.294	0.094	0.531	0.342

SEM, standard error of the mean; D, ensiling days; T, storage temperature; DM, dry matter; LA, lactic acid (g/kg DM); AA, acetic acid (g/kg DM); PA, propionic acid (g/kg DM); BA, butyric acid (g/kg DM); NH_3_-N, NH_3_-nitrogen (% total nitrogen).

a–d Means within columns with different superscript letters differ significantly from each other (p<0.05).

**Table 3:** Chemical composition of four tropical forage silages during ensiling

Items		30 days		60 days		SEM	p-value		
		
		28°C	40°C	28°C	40°C		D	T	D×T
King grass	DM	22.2^a^	22.8^a^	19.8^b^	20.3^b^	1.256	<0.001	0.433	0.046
	NDF	569^b^	606^a^	598^a^	589^ab^	3.887	0.466	0.098	0.018
	ADF	337^b^	350^ab^	363^a^	346^b^	2.233	0.039	0.621	0.010
	CP	113	118	112	113	0.949	0.124	0.212	0.399
	WSC	3.6^a^	3.3^a^	3.4^a^	2.5^b^	0.086	0.014	0.007	0.150
Paspalum	DM	25.3^b^	26.6^a^	23.9^c^	23.9^c^	6.841	<0.001	0.150	0.149
	NDF	684	698	688	695	4.339	0.967	0.274	0.671
	ADF	398	404	398	405	5.565	0.963	0.586	0.948
	CP	60.6	60.0	60.3	59.7	1.415	0.271	0.236	0.316
	WSC	3.7^a^	3.1^b^	2.8^b^	2.2^c^	0.067	<0.001	0.002	0.989
White popinac	DM	50.5^a^	50.9^a^	47.3^b^	48.1^b^	2.591	<0.001	0.639	0.234
	NDF	399	387	402	399	6.154	0.583	0.557	0.713
	ADF	234	227	227	229	3.993	0.769	0.811	0.555
	CP	268^ab^	264^ab^	275^a^	256^b^	1.935	0.991	0.019	0.083
	WSC	10.5^a^	10.5^a^	9.9^ab^	9.5^b^	0.138	0.023	0.486	0.454
Stylo	DM	29.6^b^	31.6^a^	28.7^c^	28.3^c^	3.021	<0.001	0.315	0.139
	NDF	663	616	640	619	9.471	0.603	0.113	0.507
	ADF	429	418	428	417	5.627	0.912	0.370	0.993
	CP	133	135	134	134	0.938	0.032	0.016	0.109
	WSC	3.9	4.3	4.0	3.7	0.168	0.797	0.456	0.317

SEM, standard error of the mean; D, ensiling days; T, storage temperature; DM, dry matter (% FM); NDF, neutral detergent fiber (g/kg DM); ADF, acid detergent fiber (g/kg DM); CP, crude protein (g/kg DM); WSC, water-soluble carbohydrates (g/kg DM).

a–c Means within columns with different superscript letters differ significantly from each other (p<0.05).

**Table 4:** Microbial composition of four tropical forage silages during ensiling

Items	Microorganism (log cfu/g FM)	30 days		60 days		SEM	p-value		
		
		28°C	40°C	28°C	40°C		D	T	D×T
King grass	LAB	6.2^b^	7.0^a^	7.4^a^	5.6^c^	0.089	0.429	0.013	<0.001
	Yeasts	ND	ND	ND	ND	-	-	-	-
	Molds	ND	ND	ND	ND	-	-	-	-
Paspalum	LAB	5.1^c^	6.6^b^	7.6^a^	5.1^c^	0.102	0.027	0.086	<0.001
	Yeasts	ND	ND	ND	ND	-	-	-	-
	Molds	ND	ND	ND	ND	-	-	-	-
White popinac	LAB	5.5^c^	6.2^b^	7.5^a^	4.8^d^	0.045	0.012	<0.001	<0.001
	Yeasts	ND	ND	ND	ND	-	-	-	-
	Molds	2.98	ND	ND	ND	-	-	-	-
Stylo	LAB	5.1^c^	5.3^b^	6.6^a^	3.7^d^	0.056	0.836	<0.001	<0.001
	Yeasts	ND	ND	ND	ND	-	-	-	-
	Molds	ND	ND	ND	ND	-	-	-	-

FM, fresh matter; SEM, standard error of the mean; D, ensiling days; T, storage temperature; LAB, lactic acid bacteria; ND, not detected.

a–d Means within columns with different superscript letters differ significantly from each other (p<0.05).

**Table 5:** Diversity statistics of bacterial community and abundances of the most relatively abundant bacterial genera during ensilage process

Sample	Diversity statistics				Abundance of genus (%)	
	
	OTU	Chao	Coverage	Shannon	Lactobacillus	Enterobacter
K	260	374.43	0.996	2.35	0	0.1
KL30	195	328.00	0.997	2.83	8.2	60.7
KH30	218	327.77	0.996	3.07	20.8	49.3
KL60	172	245.70	0.997	2.64	25.8	53.9
KH60	273	344.08	0.997	2.36	19.8	9.2
P	1055	1801.82	0.978	3.91	0	0
PL30	356	408.98	0.997	4.08	0.2	27.5
PH30	225	341.00	0.996	3.08	5.3	46.9
PL60	246	388.68	0.996	3.04	7.4	49.2
PH60	138	202.69	0.998	1.91	0.4	64.4
W	124	167.33	0.998	0.47	0.3	0.2
WL30	270	460.38	0.996	3.31	26.5	5.1
WH30	291	459.00	0.995	3.03	44.4	9.0
WL60	252	333.00	0.997	3.24	38.4	26.7
WH60	565	631.28	0.997	4.95	14.2	0.9
S	161	196.88	0.998	1.43	0.3	0.2
SL30	163	270.65	0.997	2.57	2.9	63.3
SH30	158	215.12	0.998	2.56	6.8	66.5
SL60	188	287.04	0.997	2.73	3.1	74.6
SH60	192	209.10	0.999	2.64	7.2	66.6

OTU, operational taxonomic units; K, king grass; P, paspalum; W, white popinac; S, stylo; L, low temperature (28°C); H, high temperature (40°C). 30, 30 days; 60, 60 days.
